# Adaptation of the yeast gene knockout collection is near-perfectly predicted by fitness and diminishing return epistasis

**DOI:** 10.1093/g3journal/jkac240

**Published:** 2022-09-09

**Authors:** Karl Persson, Simon Stenberg, Markus J Tamás, Jonas Warringer

**Affiliations:** Department of Chemistry and Molecular Biology, University of Gothenburg, 40530 Gothenburg, Sweden; Department of Biology and Biological Engineering, Chalmers University of Technology, 41296 Gothenburg, Sweden; Department of Chemistry and Molecular Biology, University of Gothenburg, 40530 Gothenburg, Sweden; Department of Chemistry and Molecular Biology, University of Gothenburg, 40530 Gothenburg, Sweden; Department of Chemistry and Molecular Biology, University of Gothenburg, 40530 Gothenburg, Sweden

**Keywords:** adaptation, evolvability, epistasis, diminishing return, yeast, *Saccharomyces cerevisiae*

## Abstract

Adaptive evolution of clonally dividing cells and microbes is the ultimate cause of cancer and infectious diseases. The possibility of constraining the adaptation of cell populations, by inhibiting proteins enhancing the evolvability, has therefore attracted interest. However, our current understanding of how genes influence adaptation kinetics is limited, partly because accurately measuring adaptation for many cell populations is challenging. We used a high-throughput adaptive laboratory evolution platform to track the adaptation of >18,000 cell populations corresponding to single-gene deletion strains in the haploid yeast deletion collection. We report that the preadaptation fitness of gene knockouts near-perfectly (*R^2^=* 0.91) predicts their adaptation to arsenic, leaving at the most a marginal role for dedicated evolvability gene functions. We tracked the adaptation of another >23,000 gene knockout populations to a diverse range of selection pressures and generalized the almost perfect (*R^2^=*0.72–0.98) capacity of preadaptation fitness to predict adaptation. We also reconstructed mutations in *FPS1*, *ASK10*, and *ARR3*, which together account for almost all arsenic adaptation in wild-type cells, in gene deletions covering a broad fitness range and show that the predictability of arsenic adaptation can be understood as a by global epistasis, where excluding arsenic is more beneficial to arsenic unfit cells. The paucity of genes with a meaningful evolvability effect on adaptation diminishes the prospects of developing adjuvant drugs aiming to slow antimicrobial and chemotherapy resistance.

## Introduction

Clonal adaptive evolution of microbial and somatic cell populations to tissues, immune defenses, and the behavior of humans, crop plants, and domesticated animals is the ultimate cause of infection and cancer. Approaches that seek to constrain the capacity of clonal cell populations to generate and transmit beneficial variation, i.e. their evolvability, have therefore attracted increasing interest (Payne and Wagner 2019). Gene products promoting evolvability are potential chemical targets whose inhibition could slow the growth of cancers and infections and delay resistance development (Ragheb *et al.* 2019).

The mechanisms underlying evolvability have been extensively explored theoretically (Conrad 1990; Alberch 1991; Houle 1992; Wagner and Altenberg 1996; Wagner 1996; Pigliucci 2008). Experimental studies have also shown the involvement of cellular processes such as DNA replication and repair that control the mutation rate (Sniegowski *et al.* 1997; Wielgoss *et al.* 2013; Lynch *et al.* 2016). However, while high mutation rates shorten the wait for selectable beneficial variants, they also increase the genetic load of deleterious mutations and their net effect on adaptation rates is therefore subject to debate (Ram and Hadany 2012). Alleles increasing population sizes, or speeding up cell divisions, generate more mutations per time unit in a cell population and thus enhance its evolvability (Olson-Manning *et al.* 2012). Sexual recombination in turn speeds adaptation by combining beneficial alleles into one genome and by freeing them from linked deleterious variants (McDonald *et al.* 2016). Genes promoting mating, outbreeding, and the frequency of meiotic cross-overs can therefore all be thought of as evolvability genes.

Imperfections in transcription, translation, and protein degradation (Elowitz *et al.* 2002; Blake *et al.* 2003) lead to macromolecular diversity in cell populations and fitness heterogeneity and may therefore enhance evolvability. Yeast expresses the self-propagating prion form [*PSI+*] of the translational termination protein Sup35 (True and Lindquist 2000) when stressed (Tyedmers *et al.* 2008), reducing the translational termination fidelity (Firoozan *et al.* 1991; Eaglestone *et al.* 1999; Lancaster *et al.* 2010; Baudin-Baillieu *et al.* 2014). Thus, the [psi−]/[*PSI+*] system serves as an evolutionary capacitor: the [psi−] state canalizes the favorable [psi−] phenotypes in the absence of stress, when no adaptation is needed, while the [*PSI+*] state is switched on in challenging environments, when adaptation is needed, and then generates novel protein variants that result from stop-codon readthrough (True and Lindquist 2000; Lancaster *et al.* 2010; Zabinsky *et al.* 2019). Chaperones such as the yeast Hsp90, GroEL in *Escherichia coli*, and Hsp-110 in *Caenorhabditis elegans* may have similar functions as evolutionary capacitators, canalizing favorable phenotypes during benign conditions by chaperoning mutated peptides into a standard protein fold, while allowing the expression of these mutations as new protein variants during stress (Rutherford and Lindquist 1998; Tokuriki and Tawfik 2009; Jarosz and Lindquist 2010; Koneru *et al.* 2021).

Finally, it has been proposed that gene networks may function as evolutionary capacitators, their robust topology masking phenotypic variation when the environment is favorable. Cells may be able to inactivate critical network nodes in adverse conditions, thereby changing the topology of networks and unmasking phenotypic variation in adverse conditions (Bergman and Siegal 2003). Around 300 yeast genes have been shown to affect phenotypic variation (Levy and Siegal 2008), with chromatin regulators being particularly important (Tirosh *et al.* 2010).

However, while it is broadly accepted that some genes can influence evolvability in the sense that they control the amount of variation that is expressed within cell populations, it remains mostly unclear whether these, or other genes, influence the adaptation dynamics. This is primarily because screening for genetic effects on adaptation rates is experimentally challenging.

## Materials and methods

### Yeast strains

The haploid BY4741 single-gene deletion collection (*MAT****a***; *his3*Δ*1*; *leu2*Δ*0*; *met15*Δ*0*; *ura3*Δ*0*; *GeneX*::*kanMX*) and its parental strain BY4741 (wild type) were used in all adaptive evolution experiments (Giaever *et al.* 2002). To construct double gene knockouts, single gene knockout strains were crossed with one of several BY4742 query strains (*MAT*α; *GeneX*::*natMX4*; *can1*Δ::*STE2pr-Sp_his5*; *lyp1*Δ; *his3*Δ*1*; *leu2*Δ*0*; *ura3*Δ*0*; *met15*Δ*0*). For gene duplication strains, single knockout lines were transformed with a centromeric plasmid (MoBY-YPR201W) containing *ARR3* (Hei Ho *et al.* 2013).

### Yeast cultivation conditions

Frozen glycerol stocks of yeast strains were recovered on YPD (Yeast Peptone Dextrose) medium supplemented with G418 (Geneticin, 200 mg/L). Wild-type cells were recovered on YPD without added G418. Except from revival of frozen stocks, yeast strains were cultivated on a Synthetic Complete medium (SC) composed of 0.14% Yeast Nitrogen Base (CYN2210, ForMedium), 0.5% NH_4_SO_4_, 0.077% Complete Supplement Mixture (CSM; DCS0019, ForMedium), 2.0% (w/v) glucose, pH buffered to 5.8 with 1.0% (w/v) succinic acid and 0.6% (w/v) NaOH and addition of 2.0% (w/v) agar for solid medium. Selective environments consisted of SC medium, but with addition of: 3 mM arsenite ([As III]; NaAsO_2_), 4 mM arsenite, 0.25 mg/L rapamycin, 400 mg/L paraquat (methylviologen; N, N-dimethyl-4-4′-bipiridinium dichloride) or 1.25 M sodium chloride (NaCl). Agar dissolved in deionized water was autoclaved and cooled to 60°C before stock solutions and stressors were added. Solid medium Singer Plus plates (Singer Instruments, UK) were cast on a solid surface by addition of 50-mL medium. Plates were allowed to dry at room temperature for 2 days before use.

All yeast strains were stored at −80°C in 20% glycerol and cultivated at 30°C. Yeast populations were subsampled and transferred to fresh plates by robotic pinning (ROTOR HDA, Singer Instruments, UK).

### Strain construction

#### Double gene deletions

Double gene deletion strains were constructed using the Synthetic Genetic Array method (Kuzmin *et al.* 2014). Query gene deletion strains, lacking *FPS1*, *ASK10*, *URA3*, *HO*, or *HIS3,* were prepared as lawns by spreading 800-µL liquid culture on YPD agar, supplemented with adenine (120 mg/L) and clonNAT (100 mg/L). Target single gene deletion strains were robotically pinned in 384 array formats on separate YPD agar plates supplemented with G418 (200 mg/L). Plates were incubated at 30°C for 2 days, ensuring sufficient growth. A 384 query strain array was generated by pinning the query strain lawn, using 384 pin pads, onto fresh YPD medium. Heterogeneous colonies containing both query and target strains were generated by pinning the target strain array on top of the YPD query strain array. Heterogeneous colonies were then mixed robotically, and incubated for 1 day at 22°C to allow mating. Mixed colonies were subsampled by pinning and subsamples were transferred to YPD agar, supplemented with G418 (200 mg/L) and clonNAT (100 mg/L) to select for *MAT***a**/α diploid zygotes, and incubated for 2 days at 30°C. The resulting array of *MAT***a**/α diploid zygotes was transferred to enriched sporulation agar plates (Kuzmin *et al.* 2014) and incubated at 22°C for 14 days to ensure a high sporulation efficiency. To select for *MAT***a** meiotic haploid progeny, sporulating colonies were subsampled and transferred to SC agar without His/Arg/Lys and without succinate buffer, but supplemented with canavanine (50 mg/L) and thialysine (100 mg/L) and with monosodium glutamic acid (MSG, 1 g/L) instead of ammonium sulfate. The array was incubated for 2 days at 30°C. The spores were then transferred to SC MSG without His/Arg/Lys, but supplemented with canavanine/thialysine/G418 (concentrations as above), and incubated for 2 days at 30°C. In the final selection step, the arrays were transferred to SC MSG without His/Arg/Lys, but supplemented with canavanine/thialysine/G418/clonNAT (concentrations as above), and incubated for 2 days at 30°C. To ensure that the resulting array consisted of haploid double gene deletion mutants, the entire array was transferred a second time to fresh plates containing the same medium.

#### Gene duplications

We constructed single gene deletion strains carrying a centromeric plasmid (MoBY-YPR201W) with an extra *ARR3* copy (Hei Ho *et al.* 2013) in a microtiter plates using the standard Lithium acetate (LiAc)/single-stranded carrier DNA/polyethylene glycol (PEG) method (Gietz and Schiestl 2007). Target strains were cultivated for 3 days on solid media in 96 array format. A transformation mix was prepared for each 96 well plate consisting of 1 M LiAc, 1.5 mL; single-stranded carrier DNA (2 g/L, denatured at 95°C for 5 min, then transferred to ice), 2 mL; plasmid (≥100 ng/well) dissolved in sterile deionized water, 1.5 mL. For each transformation, 50-µL transformation mix was added per well. Yeast cells were transferred robotically to the 96 well plate and mixed into suspension. To each transformation reaction, 100-µL PEG 3350 (50% w/v) were added and mixed. The plates were incubated at 42°C for 1.5 h. To recover the transformants, plates were centrifuged at 1,500 *g* for 10 min and the supernatant discarded. The pelleted cells were suspended by pipetting in 50-µL liquid selective media (SC-URA), 20 µL of the cell suspension were transferred to a fresh 96 well microtiter plate containing 150 µL selective media (SC-URA) and incubated at 30°C for 3 days. Transformants were stored in 20% glycerol at −80°C.

### Artificial laboratory evolution procedure

Cells were evolved in either 384 or 1,536 colony arrays arranged on top of solid medium. Deletion mutant strains, and wild-type cell populations to be used as a baseline for calling genetic effects on adaptation rates, were stored in 384 colony arrays as −80°C glycerol stocks. Stored cell populations were thawed and recovered on YPD + G418 media, leaving every fourth colony position empty. After 3 days of cultivation at 30°C on the recovery plate, recovered cell populations were subsampled and replicated 3 times onto different SC medium preculture plates (Supplementary Fig. 1). In parallel, wild-type cell populations to be used as nonevolving spatial controls on experimental plates were recovered on YPD, as 384 colony arrays, and transferred to a separate preculture plate containing SC media. The evolution of gene deletion strains and interleaved evolving wild-type controls was initiated and continued by pinning first the precultures, and then each successive evolutionary batch culture, onto stressor-containing evolution plates. After each batch cycle of evolution, evolving gene deletion strain and wild-type cell populations were transferred to stressor-containing preculture plates and preculture for growth phenotyping experiments. At the precultivation stage, we introduced nonevolving wild-type spatial controls into every fourth, previously empty, colony position and later used these to account for environmental variations within and between plates. Precultures for growth phenotyping were transferred to stressor-containing experimental plates after 72 h at 30°C. These experimental plates were used for the growth phenotyping experiments described below. We generated cycle 0 estimates of preadaptation growth by pinning cell populations from recovery plates onto SC medium precultivation plates without stressor. The wild-type spatial controls were transferred using 384 pin-pads, while all other cell transfers used 1,536 pin-pads.

### Measuring population doubling time

We tracked the growth of all cell populations expanding clonally on the experimental plates using the Scan-o-matic system (Zackrisson *et al.* 2016) version 2.2 (https://github.com/Scan-o-Matic/scanomatic/releases/tag/v2.2, last accessed 2022-09-14). Plates were maintained undisturbed without lids for 72 h in high-definition desktop scanners (Epson Perfection V800 PHOTO scanners, Epson Corporation, UK) that were placed inside dark, humid, and temperature-controlled (30°C) thermostatic cabinets. With 4 plates in each scanner, images were acquired using SANE (Scanner Access Now Easy) by transmissive scanning at 600 dpi. The plates were held in position by an acrylic glass fixture. Pixel intensity was normalized and standardized across the different scanners and experiments using a transmissive grayscale calibration strip (LaserSoft IT8 Calibration Target, LaserSoft Imaging, Germany).

The pixel intensity of the grayscale calibration strips was compared to the manufacturer’s values; this allowed normalization of variations in the light intensity of the transmission scan. Colonies were detected by the software using a virtual grid across each plate, with intersections matching the center of each colony. At the intersections, colonies and surrounding areas were segmented to determine the local background and pixel intensities. The pixel intensity was converted to total cell numbers using a predefined, independent calibration function, based on both spectroscopic and flow cytometer measurements. From this calibration, population size growth curves were obtained. The series of population size measurements were smoothed in a 2-step procedure to remove random noise variation. First, local spikes in each curve were removed by a median filter. Second, the remaining local noise was reduced by a Gaussian filter.

The growth rate at the steepest slope in each growth curve, *µ_max_*, was identified using a local regression over 5 consecutive time points, and converted into a population size doubling time. Growth curves with poor quality were automatically detected and manually inspected before exclusion. We estimated the number of cell generations passed in each growth cycle as the total number of population doublings, between the last and the first population size estimates. Population parameters were extracted as numerical values from all growth curves that passed the quality requirements. We fitted a locally estimated scatterplot smoothing (LOESS) regression to the adaptation data for each adapting population to account for technical and environmental variation, allowing estimation of the adaptation achieved at each stage of evolution for each population.

## Results

### Tracking the adaptation dynamics across >18,000 yeast gene knockout populations

**Fig. 1.**
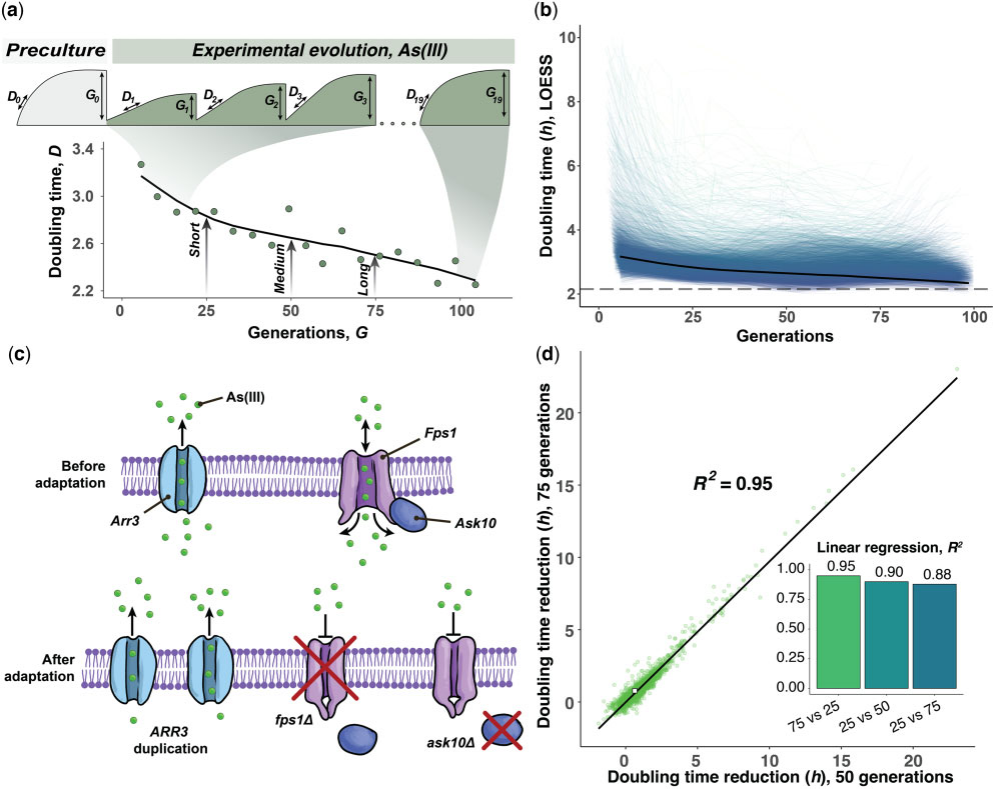
Experimental setup and phenotype extraction. a) Experimental evolution and extraction of population growth parameters. After an initial preculture cycle on basal medium, populations were evolved on the selection medium. The doubling time (*D*) and generations (*G*) in each cultivation cycle were extracted from population size growth curves. An LOESS curve was fitted to each cell doubling time adaptation curve and the adaptation achieved after 25, 50, and 75 generations were extracted from the LOESS fit. The adaptation of wild-type colonies (mean values of *n *=* *384) is shown as an example. b) Mean LOESS fitted adaptation curves of all gene deletion mutants, the wild-type adaptation is indicated with a thick grey line. The broken line shows the cell doubling time of the wild type in basal media without stress. c) Schematic illustration of the arsenite influx and efflux (top) into cells and the 3 known evolutionary solutions to adapt to arsenite by excluding it from cells (bottom). d) Comparing the cell doubling time reductions achieved by 4,639 yeast deletion strains exposed to arsenite (3 mM) over 75 (*y*-axis) and 50 generations (*x*-axis). Mean values (*n *=* *3–6) are shown, the wild type is indicated with a white square (*n *=* *384). The linear regression and the squared coefficient of linear regression are shown. *Inset:* squared linear regression coefficients when comparing cell doubling time reductions achieved over 25, 50, and 75 generations of arsenite evolution.

To probe the genetic control over clonal adaptation, we first established an adaptive laboratory evolution (ALE) framework capable of tracking the adaptation of 18,432 haploid yeast cell populations in parallel (Fig. 1a). In our ALE platform, we expanded populations from ∼50,000 to 2–4 million cells as colonies growing on a nutrient-complete synthetic agar medium, and subsampled colonies robotically after 3 days, when detectable growth had ended. We deposited cell samples on freshly made plates, repeated the batch cultivation, and then cycled each population over 19 rounds of clonal colony expansion and contraction, corresponding to 80–100 cell generations (Fig. 1a). We maintained colonies in a stable environment in bench-top scanners and estimated the population density change in each colony at 20-min intervals, based on measurements of the transmitted light (Zackrisson *et al.* 2016). We derived the adaptation for each population as the change in cell doubling time as a function of population doublings (generations; Fig. 1a). Finally, we fit an LOESS regression to the adaptation data for each population, allowing us to account for technical and environmental variation and extracting the adaptation achieved after each generation (Fig. 1a). To survey the effects of individual genes on adaptation dynamics, we evolved the collection of single yeast gene deletion strains (Giaever *et al.* 2002) clonally in the presence of arsenic in the form of trivalent arsenite (As[III]; 3 mM). Arsenite exposure at this concentration increased the cell doubling time ∼60% (from 2.27 to 3.61 h), in the average deletion strain (Supplementary Fig. 2). Arsenite is a ubiquitous selection pressure with intracellular toxicity to which cells have evolved a dedicated cellular defense system (Wysocki and Tamás 2010, 2011). Arsenite enters yeast cells primarily through the Fps1 aquaglyceroporin (Wysocki *et al.* 2001), whose activity is regulated by Ask10 (Beese *et al.* 2009; Lee *et al.* 2013), and is exported primarily by the H^+^ antiporter Arr3 (Wysocki *et al.* 1997; Fig. 1c). Natural yeast variation in As[III] resistance is explained almost exclusively by translocations and segmental duplications of the *ARR3* locus (Yue *et al.* 2017) and lab strain yeast populations exposed to high As[III] adapt either by *ARR3* amplification or by point mutations inactivating Fps1 or Ask10 (Gjuvsland *et al.* 2016). This reinstates arsenite homeostasis by excluding As[III] from cells and returns the cell doubling time to near prestress levels (Gjuvsland *et al.* 2016). Because these solutions are rapidly encountered even at relatively moderate population sizes, As[III] adaptation is swift and little afflicted by chance variations, making it an ideal testbed for gene effects on adaptation dynamics. We measured the arsenite adaptation of 4,639 yeast populations, corresponding to all single-gene deletions that were viable in the presence of 3 mM arsenite. Each gene deletion was represented by 3–6 replicate populations, allowing us to account for much of the mutational randomness and measurement error. The 384 replicate colonies of the wild-type control achieved 53.6% (cell doubling time) of their final adaptation within the first 25 generations; after that, their adaptation plateaued. They completed only 18.7% of their last adaptation after 75 generations (Fig. 1b). This wild-type pattern of adaptation was also shared by the vast majority of faster and slower adapting gene knockouts, and most genes did not measurably affect As[III] adaptation kinetics (Fig. 1b). A substantial minority of gene knockouts adapted faster than the wild type, with improvements distributed along a continuum from marginal to very large (Fig. 1b). In contrast, only a few adapted substantially slower than the wild-type adaptation, reflecting the observation that few genes benefited evolvability appreciably. To reduce the data dimensionality, we focused on the adaptation achieved over shorter (25 generations), medium (50 generations), and longer (75 generations) time spans (Fig. 1a). We found the adaptation achieved by gene knockouts at these time points to predict each other well (linear regression coefficient, *R^2^* = 0.88–0.95). We therefore assume that As[III] adaptation across different time spans is dictated by essentially the same biology (Fig. 1d, Supplementary Fig. 3) and that conclusions based on the time points above can be generalized.

### Gene knockout fitness near-perfectly predicts arsenite adaptation dynamics

**Fig. 2.**
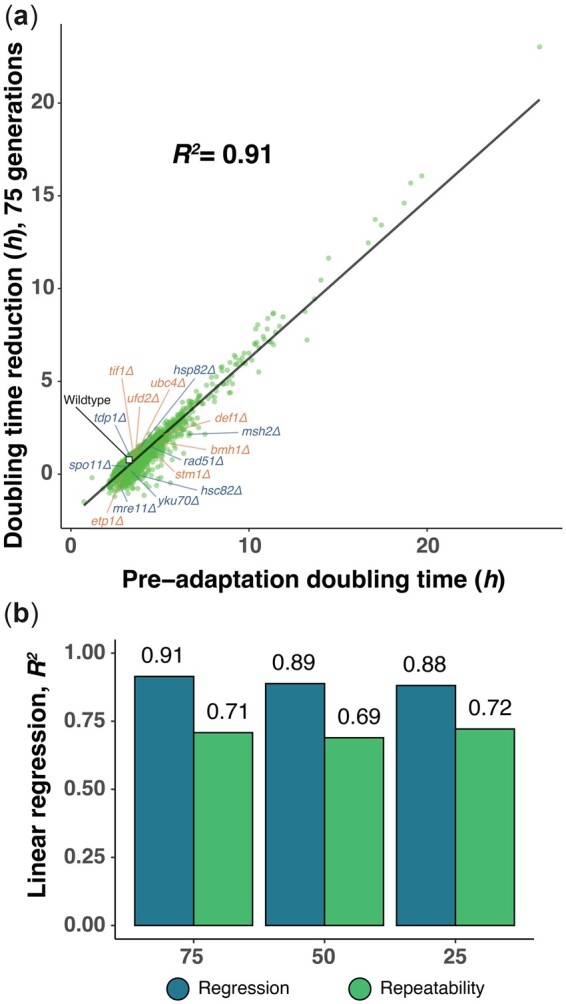
Adaptation of gene deletion strains to arsenite is near perfectly predicted by their fitness. a) The cell doubling time reduction in 4,639 yeast deletion strains exposed to arsenite (3 mM) over 75 generations as a function of their preadaptation cell doubling time. Mean values (*n *=* *3–6) are shown, the wild type is indicated with a white square (*n *=* *384). Genes traditionally held to influence the evolvability (blue) and genes known to control *ARR3* expression and thereby the physiological adjustment to arsenite (orange) are marked. The linear regression line and the squared coefficient of linear regression are shown. b) Squared coefficient of linear regression extracted when comparing the preadaptation cell doubling time to the cell doubling time adaptation over 25, 50, and 75 generations of arsenite evolution. The squared linear regression coefficient between replicate measures of the cell doubling time adaptation at these time points is shown for comparison.

The continuous decline in the adaptation rate for virtually all deletion strains suggested that their preadaptation fitness, rather than any evolvability function of the deleted gene, controls adaptation kinetics. We probed this conjecture by examining the As[III] adaptation achieved after 25, 50, and 75 generations of all 18,432 cell populations in light of their cell doubling time, as a proxy for fitness, before adaptation. Overall, the preadaptation cell doubling time predicted change in cell doubling time near perfectly, regardless of the evolutionary time span considered (linear regression coefficient, *R^2^* = 0.88–0.91; Fig. 2, a and b, Supplementary Fig. 4). The prediction accuracy generally exceeded the repeatability of single replicate measures of adaptation (linear regression coefficient, *R^2^* = 0.69–0.72), which is limited only by the measurement error, environmental variation between colony positions, and mutational randomness. The adaptation was dramatically slower for fitter gene knockouts, reflecting a diminishing return of adaptation as fitness improves (Fig. 2a, Supplementary Fig. 4). This was not due to fitter gene knockouts reaching a selection limit, dictated, for example, by cell-intrinsic constraints on growth set by ribosome production reaching a maximum, because adapting cell populations still grew slower than unstressed wild-type cell populations (Fig. 2a, Supplementary Fig. 4). Some gene knockouts adapted significantly (Students *t*-test, FDR, *q = *0.05) better (*n = *219–1,331) or worse (*n = *3–995) than expected from their arsenite fitness. Still, their deviations from the expectation for those who did were almost uniformly small (median of 0.24–0.30 h higher and 0.28–0.45 h lower) and may be due largely to environmental variation between colony positions that we have not been able to account for, rather than to intrinsic differences between gene knockouts. Consistent with this assumption, no cellular functions (yeast GO slim, Fisher’s exact test, FDR *q *>* *0.05) were enriched among these genes. Moreover, genes often suspected of influencing evolvability, such as those encoding DNA repair or protein folding functions, adapted as predicted by their fitness (Fig. 2a, Supplementary Fig. 4a). This included the Hsp90 chaperone Hsp82/Hsc82, as well as key components of the single-strand break repair (Tdp1), mismatch repair (Msh2), base-excision repair (Mre11), nonhomologous end-joining (Yku70), homologous recombination (Rad51), and meiotic recombination (Spo11). A statistical comparison also showed that cells lacking genes promoting phenotypic variation (Levy and Siegal 2008) or the mutation rate (Stirling *et al.* 2014) were not more likely to adapt slower or faster to arsenic (Fisher's exact test, *P* > 0.05) than expected by chance.

Strains lacking Ubc4, Stm1, Bmh1, Tif1, Etp1, and Ufd2, and therefore having a delayed *ARR3* expression (Ferreira *et al.* 2015; West *et al.* 2019; Romero *et al.* 2022), all adapted with the rate predicted by their preadaptation fitness. The cell doubling time extracted after 1 cycle, i.e. 72 h, in arsenite, also well predicted the adaptation achieved (linear regression coefficient, *R*^2^ = 0.77–0.83). This is consistent with the physiological adjustment to arsenite being very fast (Maciaszczyk-Dziubinska *et al.* 2010) and having no or little impact on our adaptation estimates.

We also found arsenite adaptation to be largely independent of the preadaptation doubling time of gene knockouts in the absence of arsenite (linear regression coefficient, *R^2^=*0.11). Gene knockouts with perfect growth in the absence of arsenite also sometimes vastly improved their arsenite growth. Thus, the fast adaptation of unfit gene deletion strains resulted from evolutionary rescue of arsenic specific, rather than general, growth defects.

Overall, the near-perfect predictability of the arsenite adaptation rates of gene deletion strains from their preadaptation fitness leaves virtually no room for dedicated evolvability functions.

### Fitness near-perfectly predicts gene knockout adaptation across a range of selection pressures

**Fig. 3.**
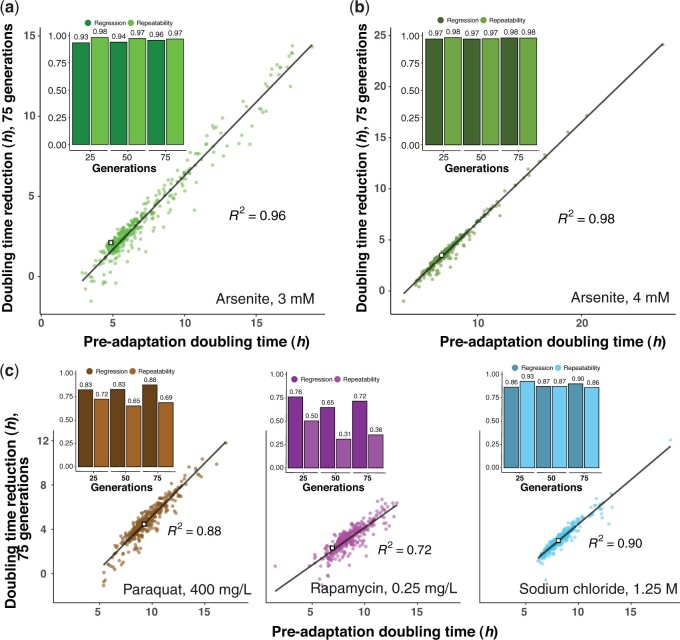
Gene deletion strain adaptation rates across a range of selection pressures. Cell doubling time adaptation achieved by 330–345 yeast deletion strains (mean of *n *=* *12–16) over 75 generations of evolution to a) arsenite 3 mM, b) arsenite 4 mM and c) paraquat 400 mg/L, rapamycin, 0.25 mg/L, and sodium chloride, 1.25 M. White squares indicate the adapting wild type. Linear regression lines and squared linear regression coefficients are shown. *Inset:* Squared linear regression coefficients extracted when comparing preadaptation cell doubling time and the cell doubling time adaptation over 25, 50, and 75 generations of evolution to the stress indicated (dark). The squared linear regression coefficient between replicate measures of the cell doubling time reduction at these time points is shown for comparison (light; single replicate repeatability).

The randomness of mutations, and the fact that new mutations can be lost due to stochastic genetic drift when still rare in populations, clearly accounts for some of the observed adaption variation between deletion strains. To reduce this source of uncertainty and further increase the confidence in conclusions, we repeated the arsenite (3 mM As[III]) ALE for 345 deletion strains, covering the complete spectrum of adaptation kinetics, at higher replication (*n = *12–16). The estimates of the adaptation dynamics of these gene knockout populations showed arsenite fitness to virtually perfectly predict all genetic variation in adaptation across the 3 evolutionary timespans considered (linear regression coefficient, *R^2^ =* 0.93–0.96; Fig. 3a, Supplementary Fig. 5). Some deletion strains deviated in adaptation from that predicted by their initial cell doubling time. Still, their deviations were small (median of 0.31–0.49 h higher, and 0.63–0.79 h lower, among significant deviations). Next, we asked whether the extraordinary predictive power of fitness on arsenite adaptation was independent of the strength of the arsenite selection. We, therefore, performed ALE on another set of 330 random deletion strains, again at high replication (*n *=* *12–16) to 4 mM As[III]. The stronger arsenite selection (wild-type cell doubling time increase 6.54 vs 4.86 h at 3 mM) forced 5 of the slowest gene deletion strains to go extinct. For the remaining 98.5% of gene deletion strains, their initial arsenite fitness again predicted essentially all arsenite genetic variance in adaptation (linear regression coefficient, *R^2^* = 0.97–0.98; Fig. 3b, Supplementary Fig. 6). Thus, the outstanding predictive power of fitness on adaptation dynamics persisted also at stronger arsenite selection. Finally, we asked whether fitness predicted the adaptation of gene deletion strains to a similar degree also under selection pressures to which cells adapt through other processes. We therefore repeated the ALE for the second set of 330 random gene knockouts, at high replication (*n *=* *12–16), under selection imposed by the redox-cycler paraquat (400 mg/L), the immunosuppressant rapamycin (0.25 mg/L), and the hyperosmotic stress inducer NaCl (1.25 M). These impair cell doubling time by targeting different aspects of yeast physiology (Supplementary Table 1), which was underscored by the low correlation in gene deletion strain growth between environments (pairwise linear regression coefficient, *R^2^<*0.01–0.02; Supplementary Fig. 7). Again, we found the initial cell doubling time of gene deletion strains in the presence of each of these stresses to predict virtually all genetic variation in their subsequent adaptation dynamics, with correlations (linear regression coefficient, *R^2^* = 0.72–0.98) approaching or exceeding that between replicated measures of adaptation (Fig. 3c). Outliers, whose adaptation was imperfectly explained by the initial fitness, were few and their deviations from the predicted adaptation were small. We compared the significant adaptation outliers at the 75 generations time point across selection pressures, and found no statistical overlap (Fisher’s exact test, *P > *0.05) among fast adapting gene knockouts. However, gene deletion strains adapting slowly were more often shared between arsenite and NaCl (14 shared vs 3 expected, Fisher’s exact test, *P *=* *7.5e−08; Supplementary Fig. 9) than predicted by chance, likely reflecting similarities in how cells adapt to arsenite and osmotic stress. Supporting this, adaptation to both arsenite and NaCl was slow in the absence of the arsenite importer and osmo-regulator *fps1*Δ, likely as a result of a shared lack of access to beneficial mutations in *FPS1*. Against expectations (Fisher's exact test, *P = *0.0095), cell populations lacking Mnn4, Irc15, Atg11, and Ygr064w adapted also to paraquat, (Supplementary Fig. 9), potentially implying that these proteins have broader roles in evolvability. Again, cells lacking key DNA repair and protein folding genes adapted to all selection pressures as predicted from their preadaptation fitness.

Stress adaptation generally occurred at the cost of a fitness loss in the absence of the stressor (Supplementary Fig. 8); underscoring that the adaptation is not to the background growth medium. Overall, the results give substantial confidence to the assessment that gene products that impact substantially on the adaptation rates of clonal yeast populations are rare.

### Diminishing return epistasis dictates yeast arsenite adaptation

**Fig. 4.**
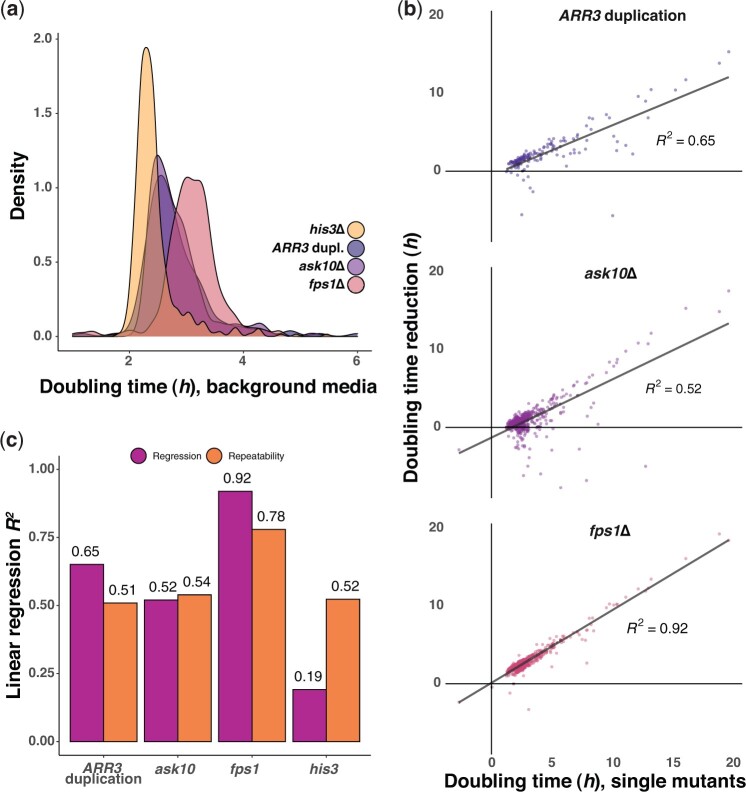
Diminishing return epistasis dictates yeast arsenite adaptation rates. a) Density distribution of cell doubling times of different gene deletion strain also missing *ASK10* (*n *=* *468) or *FPS1* (*n *=* *468) or having an extra *ARR3* copy (*n *=* *140) in the no stress background media. The same deletion strains also missing the neutral *HIS3* (*n *=* *468) are shown as controls. Means of *n *=* *6–612 replicates were used. b) Arsenite (3 mM) specific cell doubling time reductions when removing *ASK10* or *FPS1*, or inserting an extra *ARR3* copy, in different gene deletion strains (*y*-axis), compared to the cell doubling time in arsenite of these gene deletion strains (*x*-axis). Linear regressions and their squared regression coefficients are indicated. c) Squared linear regression coefficients between the arsenite (3 mM) specific cell doubling time reductions when removing *ASK10* or *FPS1*, or inserting an extra *ARR3* copy in different deletion strains and the cell doubling times, on arsenite, of these gene deletion strains. The squared linear regression coefficients between replicate measures of the cell doubling time reductions are shown for comparison (single replicate repeatability).

Adaptation is expected to decline with increasing fitness, if strongly positive mutations are few, rapidly fixate and become depleted (Orr 1998, 1999; Barton 1998). However, recent studies have provided strong support for an alternative explanation for adaptation slowing with increasing fitness: many beneficial mutations are less beneficial in fitter backgrounds (Chou *et al.* 2011; Khan *et al.* 2011; Couce and Tenaillon 2015; Miller 2019). To test the extent to which the latter hypothesis can explain the slower arsenite adaptation, we reconstructed the 3 mutation types that account for almost all arsenite adaptation in wild-type cells, *ARR3* duplication and *FPS1* and *ASK10* loss, in gene knockout strains covering a broad range of cell doubling times in the presence of arsenite. We introduced complete *FPS1* and *ASK10* gene deletions into each of 464 gene knockouts strains by mating, meiosis, and sporulation (Kuzmin *et al.* 2014) and measured their cell doubling time on 3 mM As[III] in the presence and absence of Fps1 and Ask10. *FPS1* deletion, and in some cases *ASK10* deletion, had a negative impact on cell growth in the absence of arsenite, which varied depending on the genetic background (Fig. 4a). This likely reflects the importance of cells being able to export glycerol through the Fps1 channel to maintain osmotic homeostasis (Luyten *et al.* 1995; Tamás *et al.* 1999). We accounted for these effects by comparing the doubling time of each strain in the absence and presence of arsenite and then extracting the cell doubling time effect of *FPS1* and *ASK10* deletion, respectively, on this specific measure of arsenite resistance. Overall, loss of *FPS1* conferred greater arsenite resistance than did *ASK10* loss (mean of 3.0 vs 1.0 h, Fig. 4b), consistent with the fact that Fps1 regulation involves other proteins besides Ask10 (Thorsen *et al.* 2006; Mollapour and Piper 2007; Beese *et al.* 2009; Lee *et al.* 2013; Ahmadpour *et al.* 2016). However, both Fps1 and Ask10 loss conferred much stronger benefits to arsenite sensitive than to arsenite resistant gene knockouts. In fact, the increase in arsenite resistance due to either Fps1 or Ask10 loss could be well predicted (linear regression coefficient, *R^2^* = 0.52–0.92) by the arsenite fitness of the strain into which the mutations were introduced. For example, removal of Fps1 was highly beneficial in strains lacking the transcription factors Yap1 (regulator of oxidative stress response) and Rpn4 (regulator of proteotoxic stress response; Rathod *et al.* 2018), which both are key to cells maintaining fitness on arsenite, while having much smaller effects on strains with unperturbed arsenite homeostasis (Fig. 4, b and c). We validated that the lesser impact of arsenite adaptive mutations in fitter backgrounds is not a property specific for changes to the Fps1 system by also reconstructing the Arr3 duplication in a subset of the gene deletion strains. We thus introduced an extra *ARR3* gene, carried on a single-copy plasmid, into 140 deletion strains and estimated the beneficial effect of this mutation on arsenite resistance. The pattern of a diminishing return of the *ARR3* duplication in more arsenite resistant deletion strains was abundantly clear (linear regression coefficient, *R^2^* = 0.65; Fig. 4, b and c). Overall, the power of the doubling time of deletion strain to predict the arsenite resistance conferred by introducing an Fps1 or Ask10 loss, or Arr3 duplication into this strain was high, again approaching or exceeding the capacity of replicated measures of mutation effects to predict each other (Fig. 4c). Thus, diminishing return epistasis well accounted for the variation in the effect size of arsenite beneficial mutations across deletion strains, with remaining variation likely explained by measurement error, environmental variation, or the emergence of random background mutations during the construction process. We conclude that excluding arsenite from cells through *FPS1* or *ASK10* loss-of-function mutations or through *ARR3* duplication is more beneficial if cells have poor arsenite fitness. Thus, the near-perfect predictive power of fitness on arsenite adaptation dynamics is explained by diminishing return epistasis.

## Discussion

### Evolvability genes have no meaningful role in the adaptation of haploid yeast populations

We tracked the arsenite adaptation of almost all viable single-gene deletions in the most common yeast lab strain background. We showed that essentially all genetically encoded variation in adaptation dynamics could be explained by their preadaptation variation in fitness, with less fit gene deletion strains adapting much faster than fitter. Using large subsets of gene deletion strains and high replication, we showed that this conclusion holds also at stronger selection and across a variety of biologically distinct selection pressures. The tendency for adaptation to decline with increasing fitness should come as no surprise and has been reported before, in smaller-scale studies on virus, bacteria, and yeast (MacLean *et al.* 2010; Couce and Tenaillon 2015; Jerison *et al.* 2017; Lukačišinová *et al.* 2020). What is remarkable in this more exhaustive study is the capacity of fitness to explain virtually all heritable variation in the adaptation kinetics: it leaves minimal room for dedicated evolvability functions in gene products to have had a meaningful impact on adaptation rates. This is further underscored by the fact that strains lacking gene products argued to have such roles, such as functions controlling how fast novel genetic variation emerges in cells or penetrates as phenotypic variation, adapted with almost precisely the speed predicted by their fitness. Clearly, there are limits to the extent to which these findings should be generalized. At least in the case of arsenite, our ALE populations operated in a strong selection, strong mutation regime (Sniegowski and Gerrish 2010). The abundant access to strongly beneficial arsenite resistance mutations in *FPS1*, *ASK10*, and *ARR3* means that these solutions invariably will be found very rapidly. Hence, elevations of the mutation rates, by e.g. removal of DNA replication and repair components, will do little to speed up adaptation (Gjuvsland *et al.* 2016). In smaller populations, or populations with access to fewer strong mutations, the mutation rate will be a stronger limiting factor on the adaptation rate and it cannot be excluded that elevating the mutation rate, e.g. by decreasing the DNA repair fidelity, could have a greater beneficial effect on adaptation in such populations (Sniegowski and Gerrish 2010). Second, adaptation in haploid cell populations may not perfectly capture the effects of evolvability factors on adaptation rate in diploid cell populations. In diploid cell populations, recessive variants, such as most loss-of-function mutations, can drive clonal adaptation only if first converted into homo- or hemizygotic states (Vázquez-García *et al.* 2017). Evolvability factors that promote homo- or hemizygosity, such as those inducing gene conversion, chromosome segment deletions or nonreciprocal translocations, may affect adaptation rates in diploid cell populations that we are unable to capture. Likewise, as our populations are asexual, we fail to capture any evolvability effects of mating or inbreeding or of genes increasing meiotic recombination rates. Finally, transformation or conjugation promoting factors that enhance the horizontal transmission of genes, which at least in bacteria can have large effects on adaptation rates (Graf *et al.* 2019; Alalam *et al.* 2020) are overlooked here. Bearing these caveats in mind, our finding that the >4,600 probed genes possessed no functions with a substantial impact on adaption rate is nevertheless quite remarkable and calls for caution when considering the evolutionary importance of evolvability in general. More specifically, it calls into question the idea that natural selection has acted extensively on living systems to promote the establishment of dedicated evolvability functions. And in terms of human health, it diminishes the prospects of such genes becoming valuable targets for drugs that are given together with antimicrobials or chemotherapeutics to slow resistance development.

### Global diminishing return epistasis dictates arsenite adaptation dynamics

We could explain the slower arsenite adaptation in fitter gene deletions by a diminishing return epistasis. Benefits of excluding arsenite from the cell, through loss-of-function mutations in the Fps1 arsenite importer or its positive regulator Ask10 or duplications of the arsenite efflux protein Arr3, continuously decreased with increasing fitness of the deletion strains in which these mutations were reconstructed. The smaller mutation effect sizes were evident in fit strains lacking a wide variety of gene functions, as well as in wild-type cells. They are therefore not reflections of modular epistasis (Wei and Zhang 2019) within the arsenite efflux or influx systems, but of a global epistasis where the effects of Fps1 loss, Ask10 loss, and Arr3 duplication depend on a very broad range of other variants (Kryazhimskiy *et al.* 2014). Interpreted within the context of Fps1, Ask10, and Arr3 function, such a global epistasis makes perfect biological sense: if arsenite is effectively excluded from cells, through a dramatically reduced influx (Fps1 and Ask10) or increased efflux (Arr3), it becomes irrelevant what other variants affecting arsenite homeostasis that present in a genome because their effects all depend on arsenite being present inside cells (Fig. 1c). Other gene products could conceivably affect arsenite uptake, with e.g. exported glutathione that binds extracellular arsenite and prevents its entry (Thorsen *et al.* 2012), and a small amount of arsenite entering cells through hexose transporters (Liu *et al.* 2004). But their small effects on arsenite resistance, together with the high rate of loss-of-function mutations in Fps1 and Ask10 and of Arr3 duplications, means that the latter almost invariably will drive arsenite adaptation in populations matching the size of our ALE colonies (Gjuvsland *et al.* 2016).

Diminishing return epistasis may not always be the sole genetic determinant of adaptation kinetics. Tumors with an inactivated P-glycoprotein drug efflux pump, the normal site for resistance mutations to some chemotherapeutics, adapt slowly to these treatments, reflecting a more specific genetic interaction (Binkhathlan and Lavasanifar 2013). Similarly, disrupting a drug efflux pump can slow the adaptation of *E. coli* populations exposed to antibiotics by shifting them onto evolutionary paths where some mutations reduce the effect size of key resistance mutations (Lukačišinová *et al.* 2020). Nevertheless, both theoretical (Kryazhimskiy *et al.* 2009, 2014; Perfeito *et al.* 2014; Vaishnav *et al.* 2022) and smaller-scale studies in bacteria (MacLean *et al.* 2010; Chou *et al.* 2011; Khan *et al.* 2011; Wang *et al.* 2016), virus (Levy and Siegal 2008; MacLean *et al.* 2010; Rokyta *et al.* 2011), yeast (Kryazhimskiy *et al.* 2014; Wei and Zhang 2019) and multicellular fungi (Schoustra *et al.* 2016) support a strong role of diminishing return epistasis in adaptation. Our findings underscores that the power and generality of the diminishing returns paradigm indeed are immense.

### Concluding remarks

Accurate tracking of adaptation in cell population ultimately rests on the precise counting of cells. However, counting cells at sufficiently high resolution and with sufficiently high accuracy in tens of thousands of evolving cell populations is challenging. This is primarily because light transmission through a cell population, the standard proxy for cell density, does not scale linearly with the population size, and unlike in small-scale experiments, this cannot be solved by continuously diluting populations (Warringer and Blomberg 2003). This gives rise to large measurement errors for both cell division times and the number generations based, giving rise to substantial confounding effects. Our ALE platform, which relies on the Scan-o-matic system, use built-in calibration functions and local regression to translate the transmitted light to actual population size (Zackrisson *et al.* 2016). With the caveat that the calibration functions need to be adjusted to account for the light scattering and absorbing properties of the specific cell type, the ALE platform is suitable for a broad range of microorganisms and eco-evolutionary questions. In that sense, it may help usher areas of evolutionary biology that previously have only been amenable to moderate-scale studies into the realm of high-throughput experimentation.

## Supplementary Material

jkac240_Figure_S1

jkac240_Figure_S2

jkac240_Figure_S3

jkac240_Figure_S4

jkac240_Figure_S5

jkac240_Figure_S6

jkac240_Figure_S7

jkac240_Figure_S8

jkac240_Figure_S9

jkac240_Supplemental_Material

## Data Availability

The authors declare that all data supporting the findings of this study are available within the paper as Supplemental Information Data 1–7, which are available in Mendeley Data repository at https://data.mendeley.com/datasets/r5kz3kj6f2/1 (accessed 2022 September 14). All stored unique strains and stored populations generated in this study are available from the Lead Contact without restriction. Supplemental material is available at G3 online.
