# Supplementary figures and images for: Adaptation of the yeast gene knockout collection is near-perfectly predicted by fitness and diminishing return epistasis
Source: G3 (Bethesda). 2022 Sep 9;12(11):jkac240. doi: 10.1093/g3journal/jkac240 (PMC9635671; doi:10.1093/g3journal/jkac240)

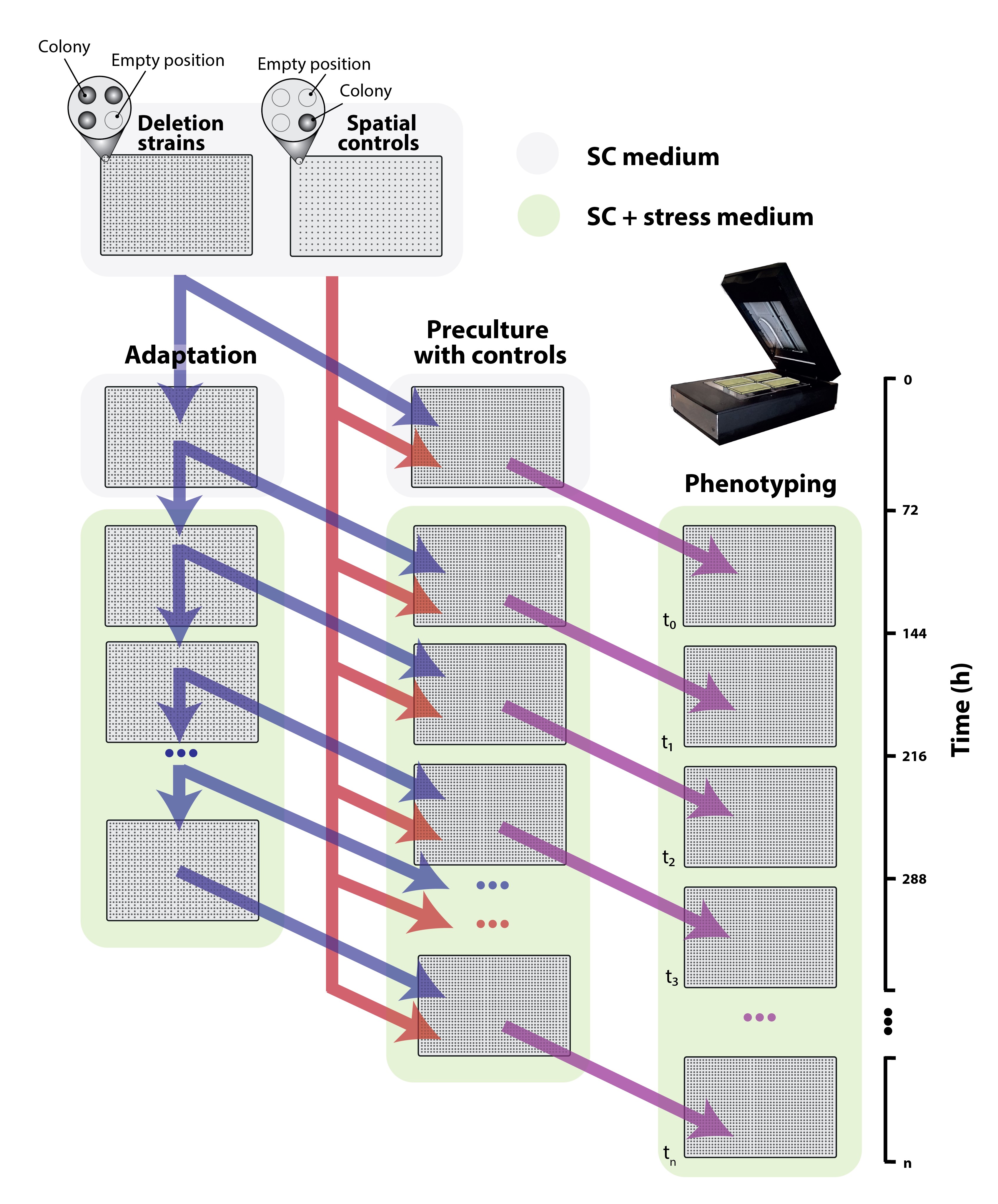

Supplement: jkac240_Figure_S1 [file jkac240_figure_s1.jpeg]

As[III] Concentration, mM

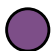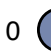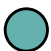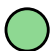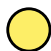

0 1 2 3 4

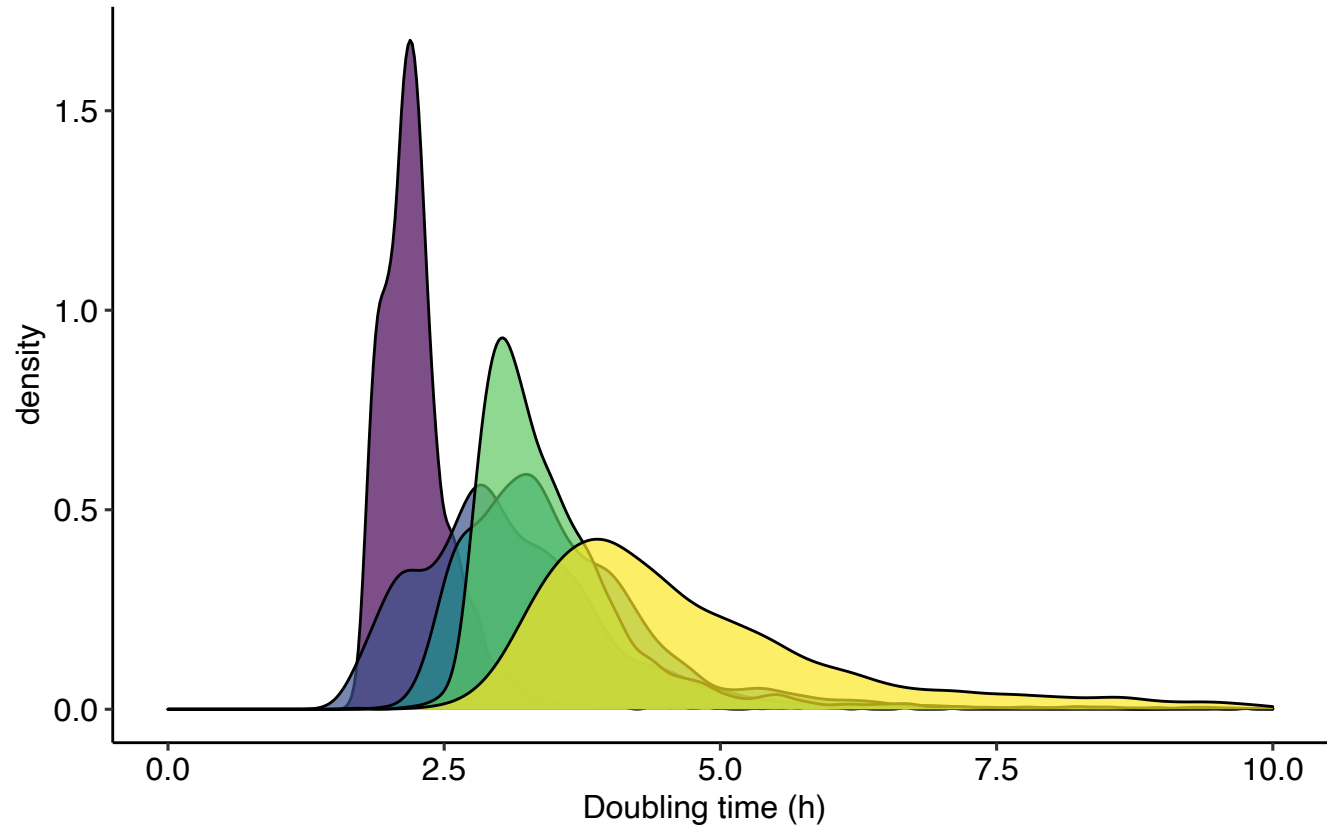

Supplement: jkac240_Figure_S2 [file jkac240_figure_s2.pdf]

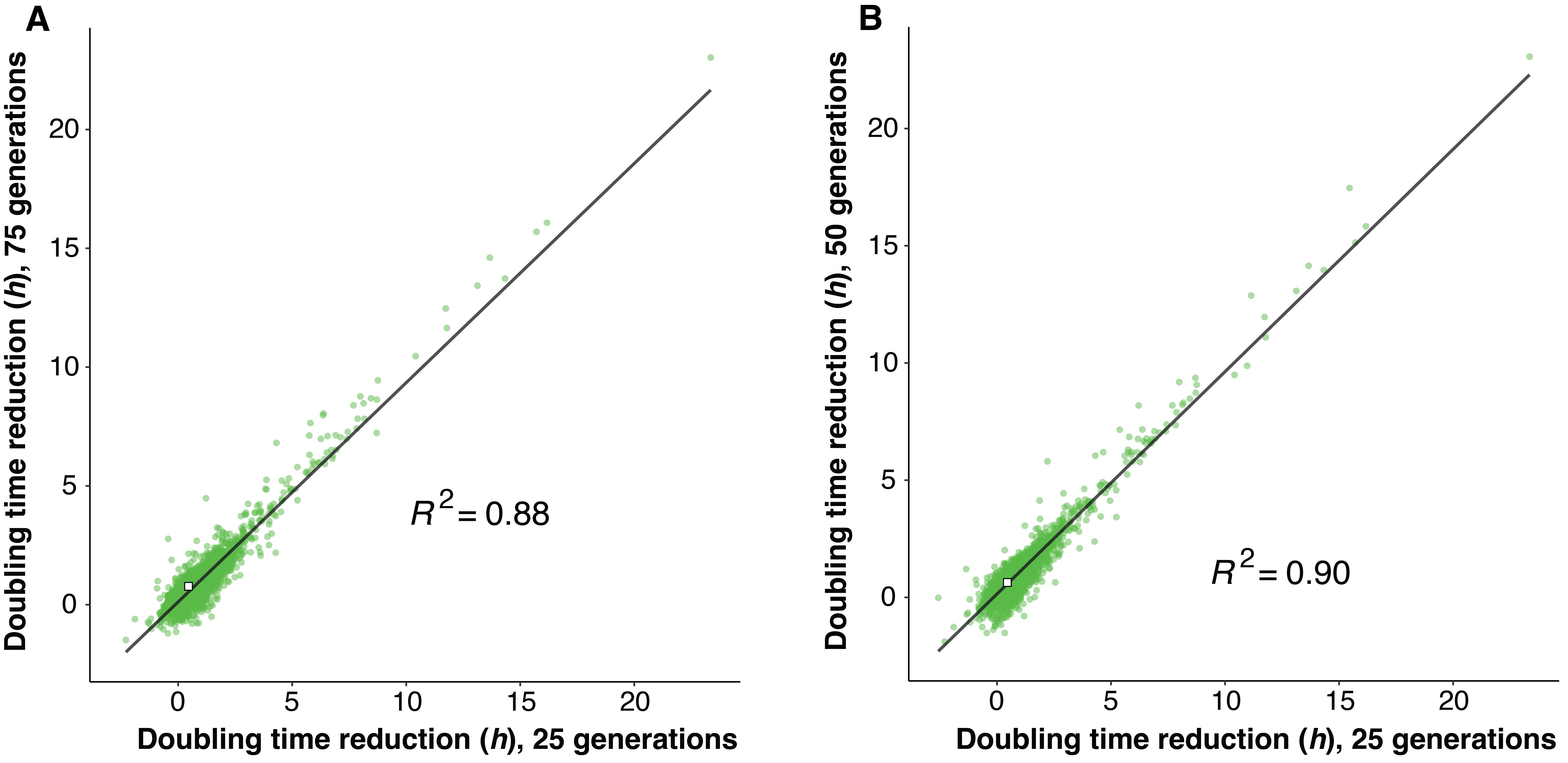

Supplement: jkac240_Figure_S3 [file jkac240_figure_s3.jpeg]

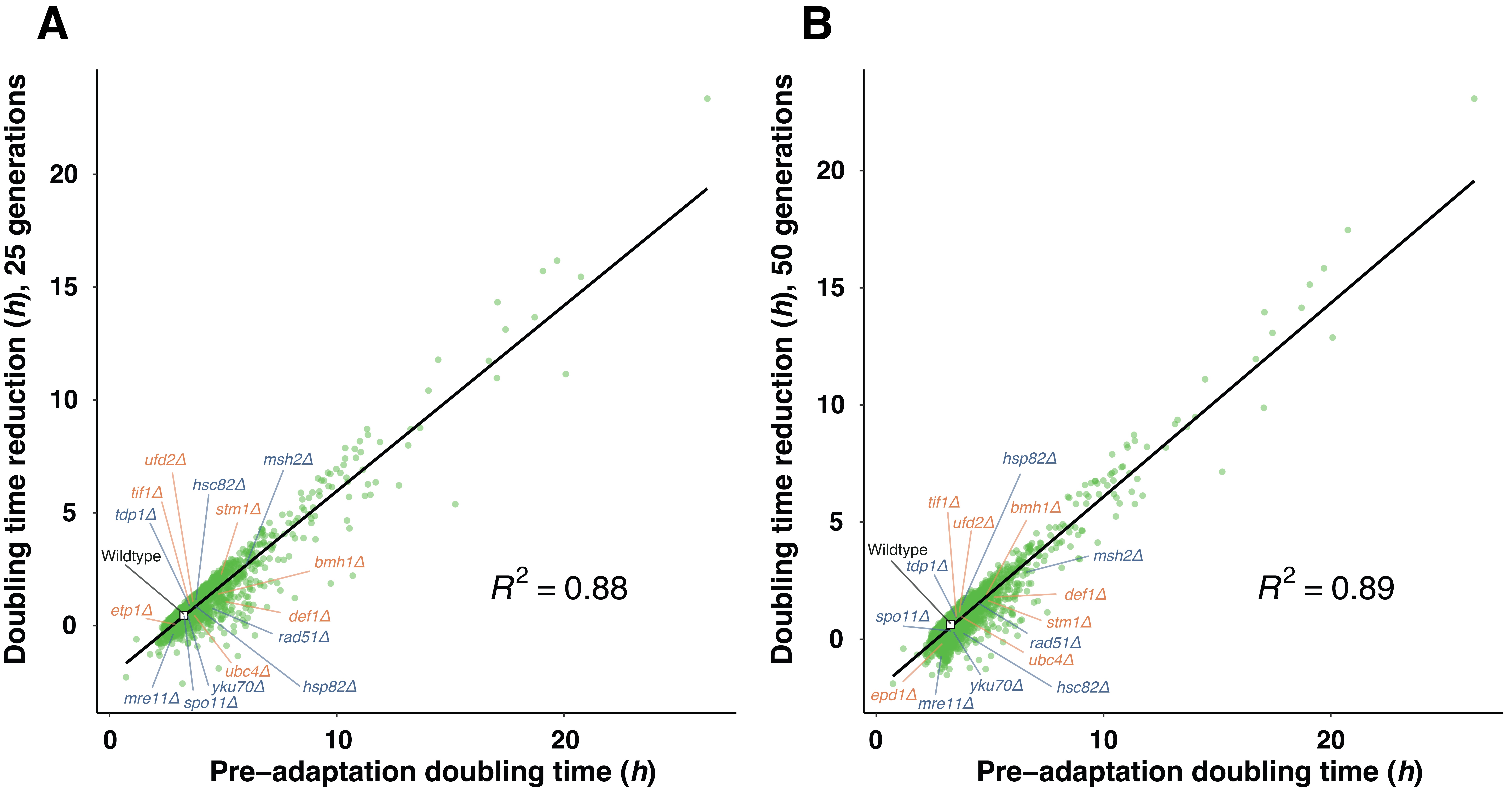

Supplement: jkac240_Figure_S4 [file jkac240_figure_s4.jpeg]

**A**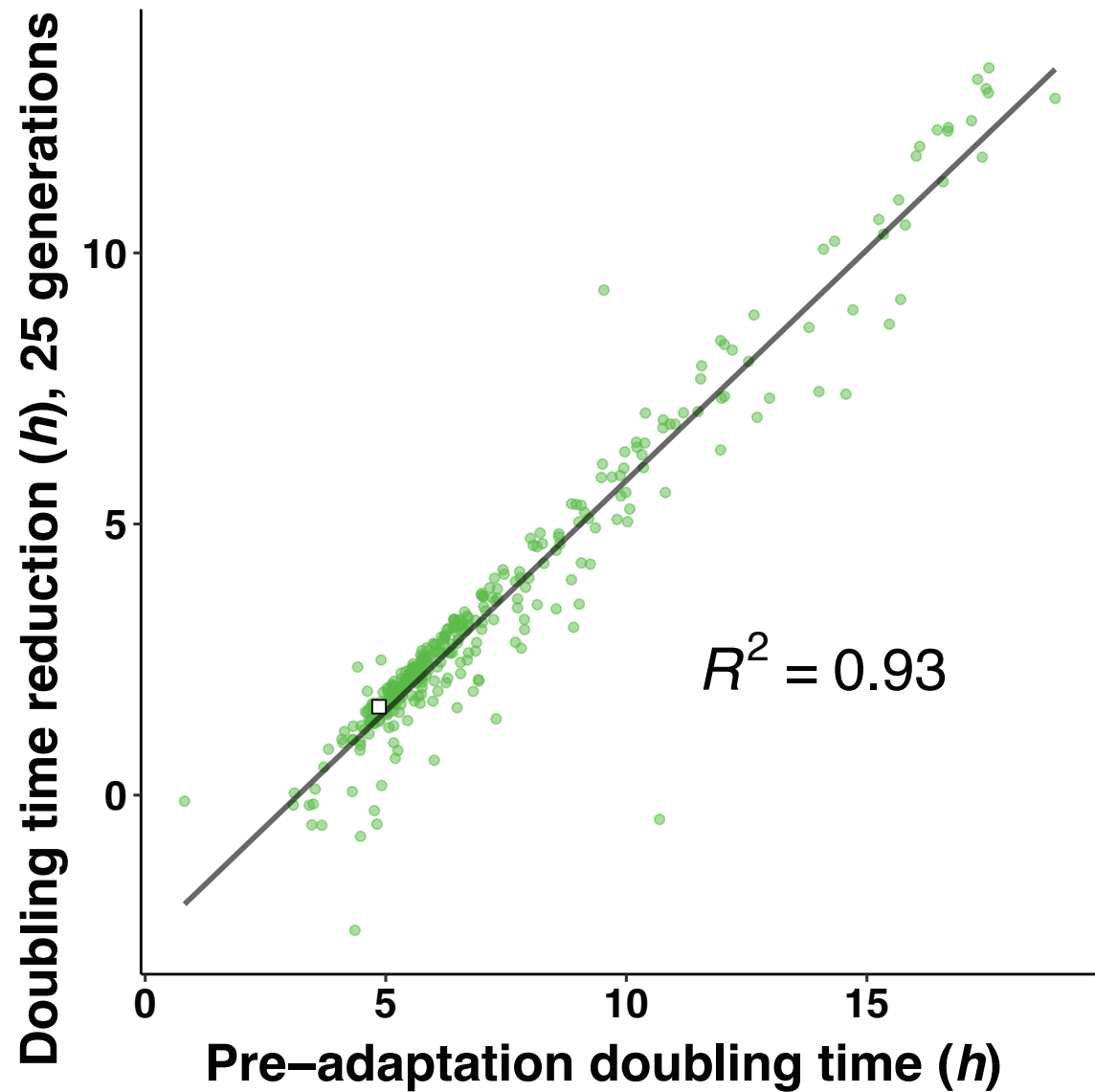**B**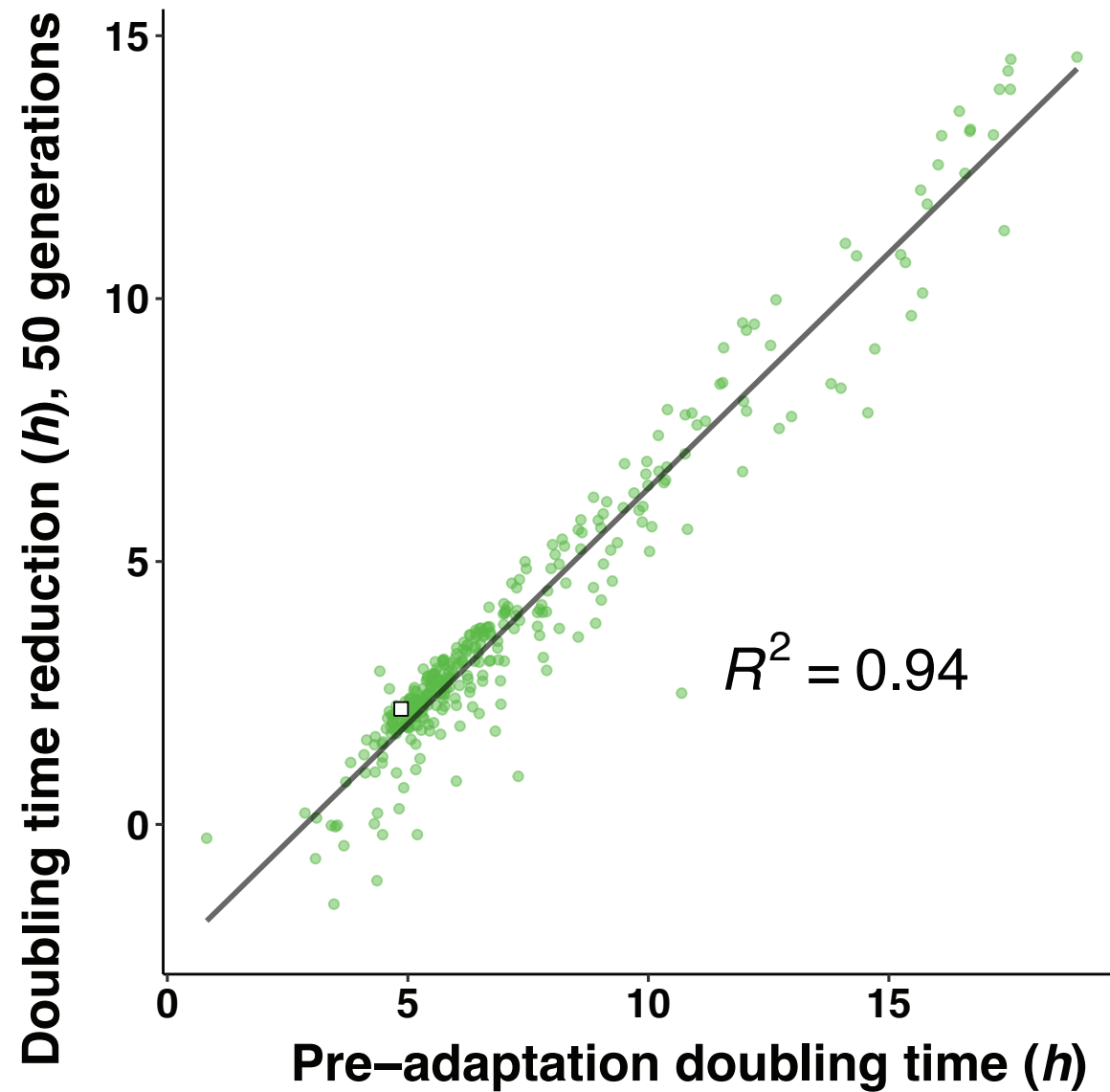

Supplement: jkac240_Figure_S5 [file jkac240_figure_s5.pdf]

**A****Doubling time reduction ( $h$ ), 25 generations**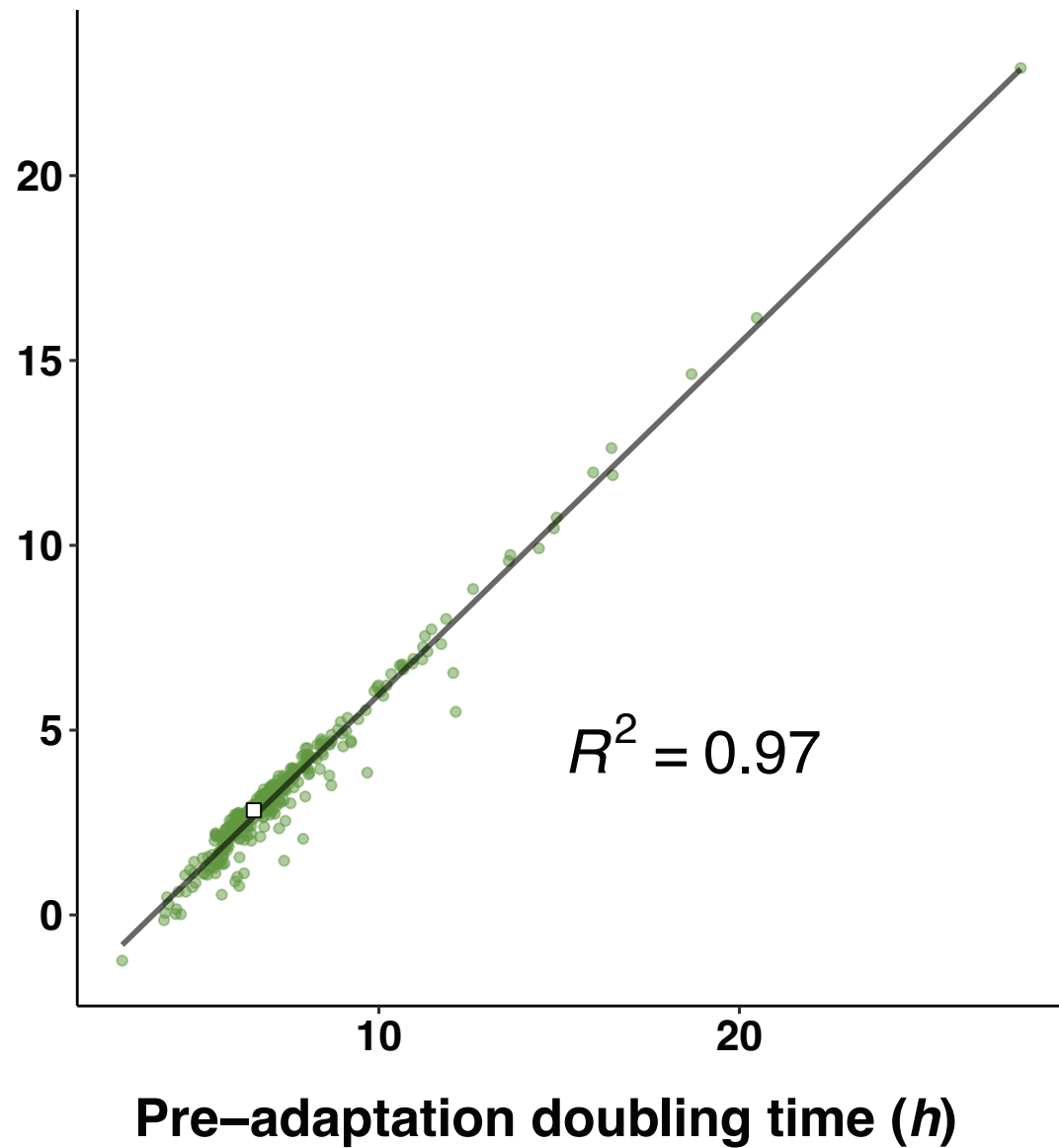**B****Doubling time reduction ( $h$ ), 50 generations**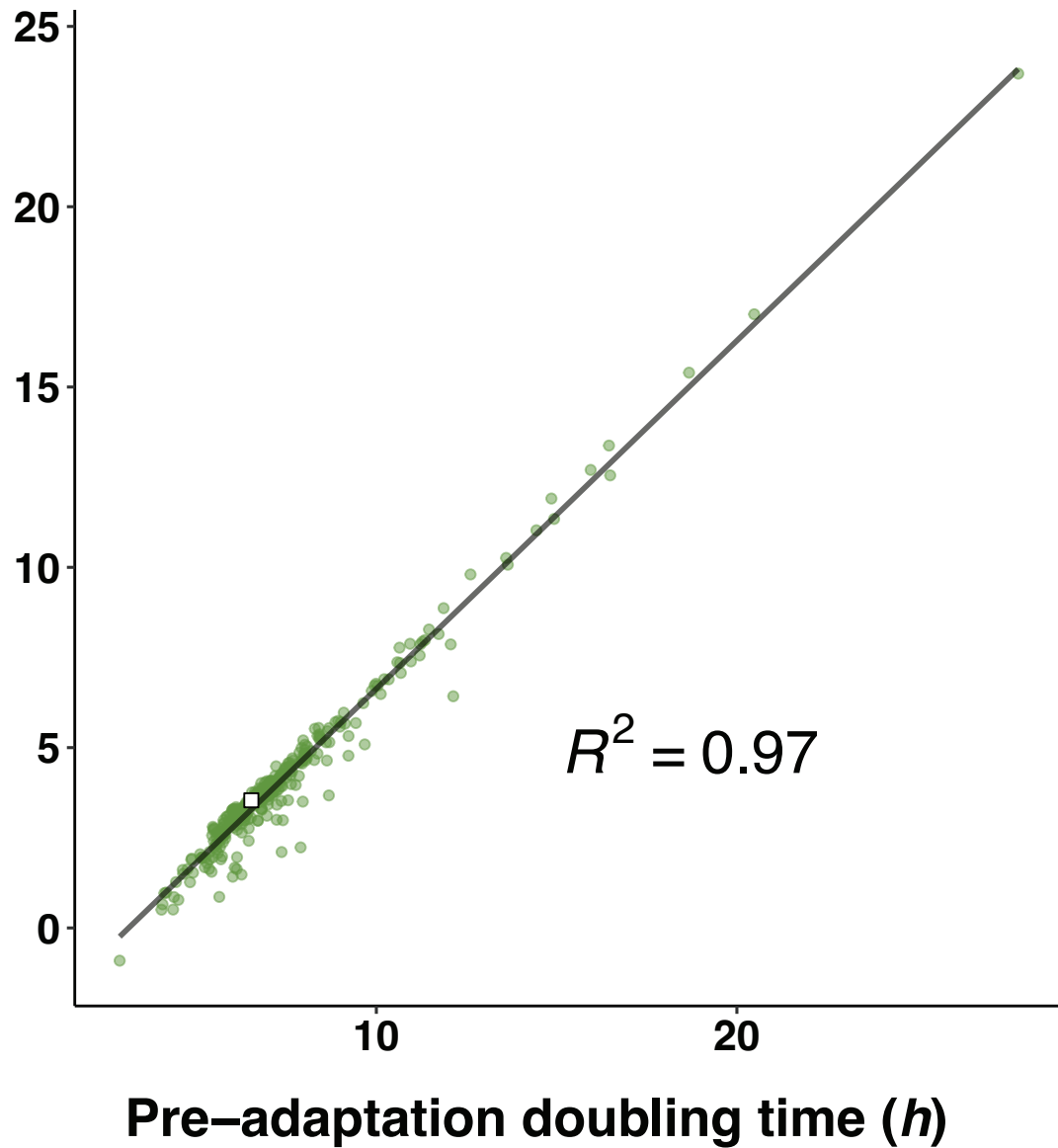

Supplement: jkac240_Figure_S6 [file jkac240_figure_s6.pdf]

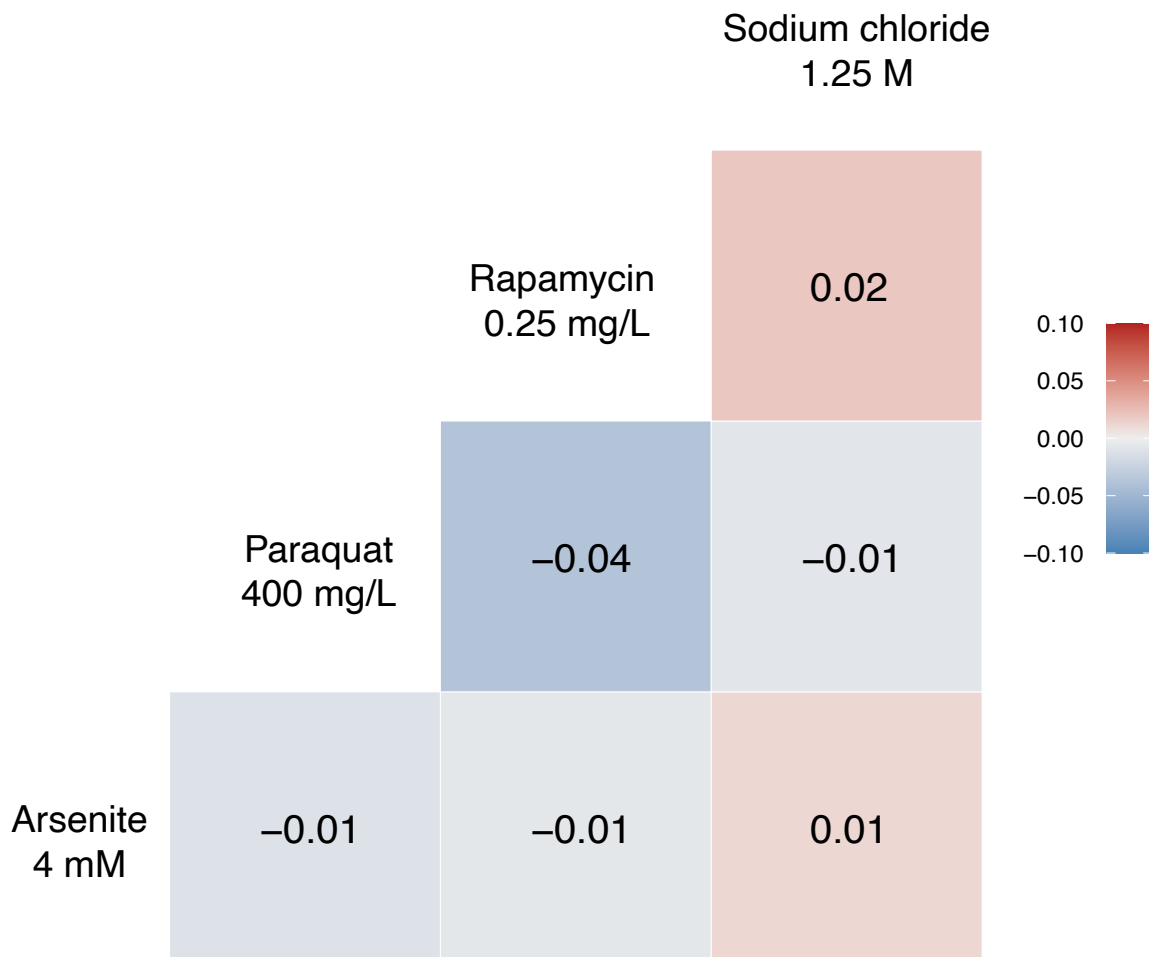

Supplement: jkac240_Figure_S7 [file jkac240_figure_s7.pdf]

Doubling time increase after adaptation

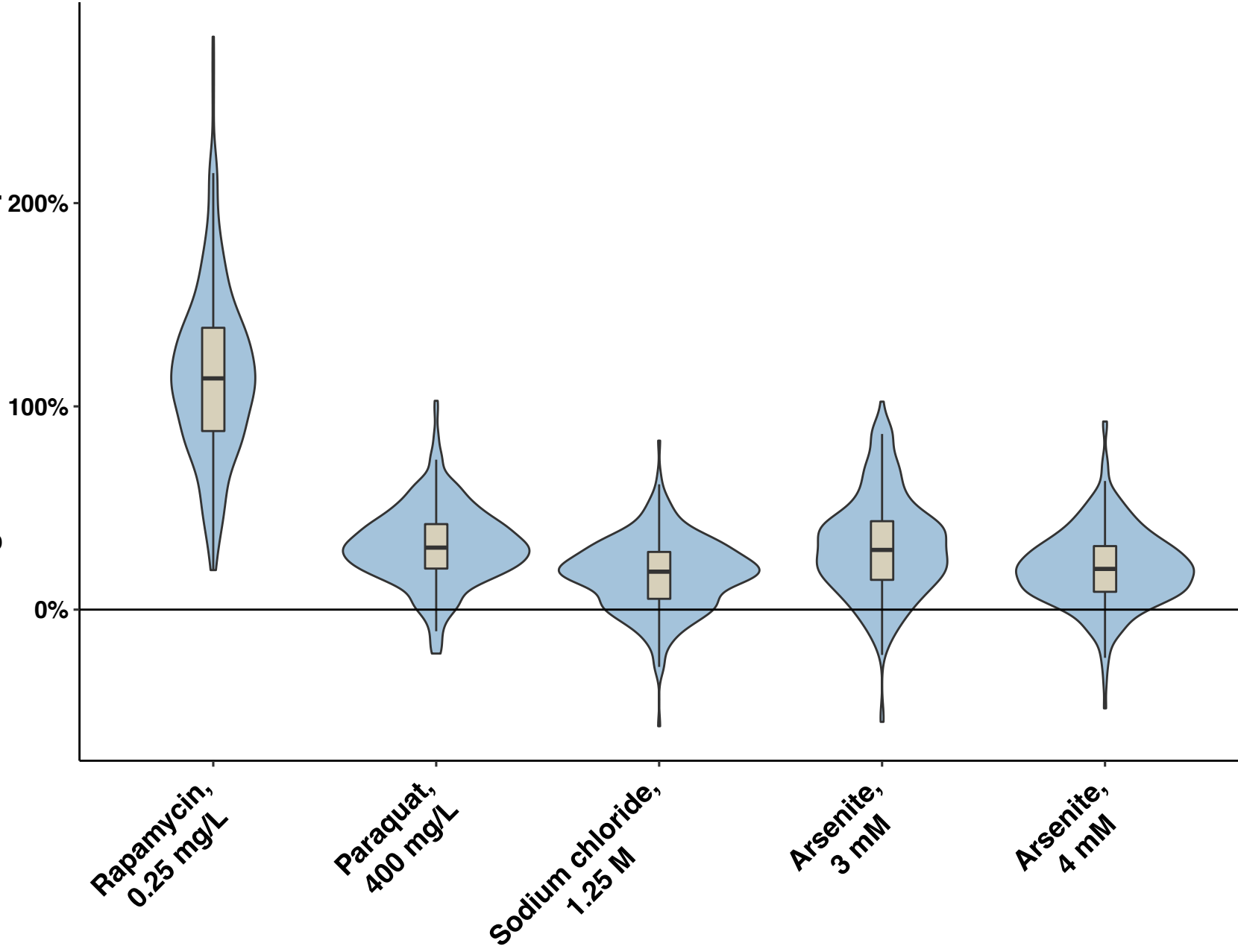

Supplement: jkac240_Figure_S8 [file jkac240_figure_s8.pdf]

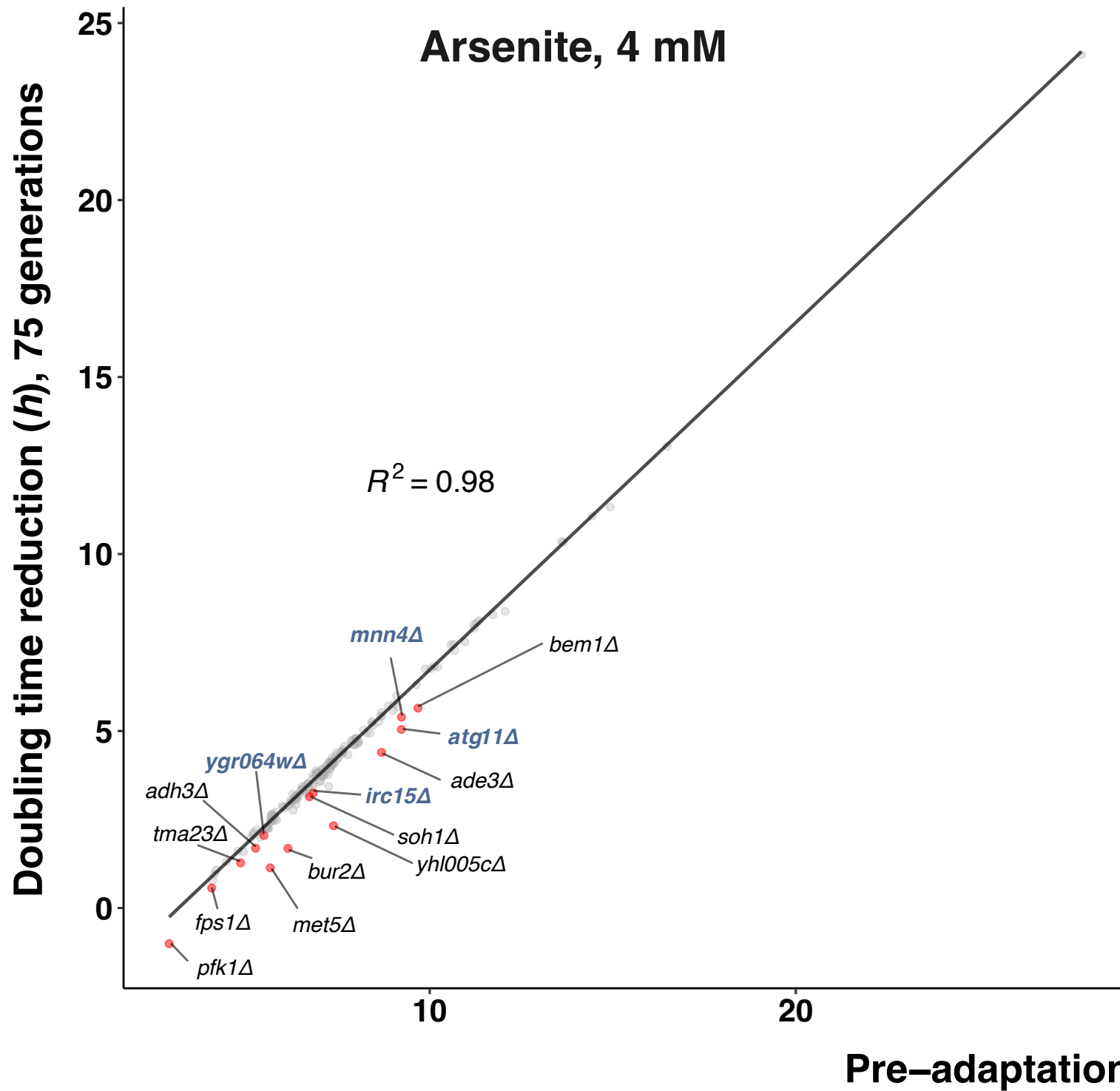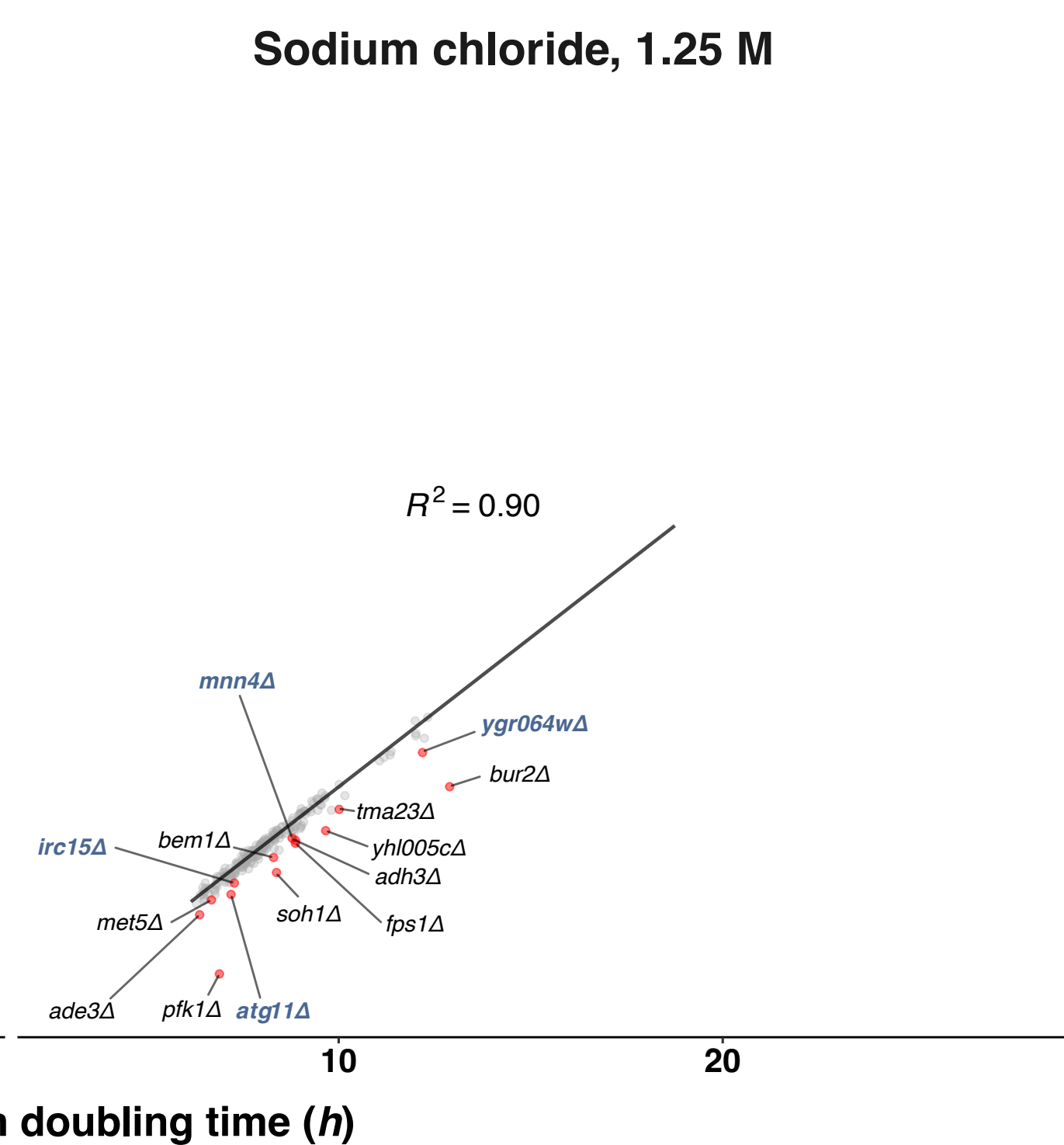

Supplement: jkac240_Figure_S9 [file jkac240_figure_s9.pdf]
