# Supplementary material for: Adaptation of the yeast gene knockout collection is near-perfectly predicted by fitness and diminishing return epistasis
Source: G3 (Bethesda). 2022 Sep 9;12(11):jkac240. doi: 10.1093/g3journal/jkac240 (PMC9635671; doi:10.1093/g3journal/jkac240)
Supplement: jkac240_Supplemental_Material [file jkac240_supplemental_material.docx]

**Supplemental Material**

**Supplemental dataset descriptions**

**Data S1:** reports dose-response growth data for gene deletion strains exposed to different degrees of arsenite stress. **Data S2:** reports growth data, before LOESS fitting, for the complete collection of BY4741 gene deletion strains during adaptive evolution in 3 mM of arsenite. **Data S3:** reports growth data, after LOESS fitting, for the complete collection of BY4741 gene deletion strains after 25, 50 and 75 generations of adaptive evolution in 3 mM of arsenite. **Data S4:** reports growth data, before LOESS fitting, of BY4741 gene deletion strains during adaptive evolution across a range of selection pressures. **Data S5:** reports growth data, after LOESS fitting, of BY4741 gene deletion strains after 25, 50 and 75 generations across a range of selection pressures. **Data S6:** reports growth data, of BY4741 gene deletion strains on background media before and after adaptation to a range of selection pressures. **Data S7:** reports growth data, of single and double mutation strains in Synthetic complete medium, and arsenite, 3 mM.

**Figure S1. Details of the experimental design of ALE experiments.** 1152 colony arrays of single gene deletion strains and wild type controls were organized in a 1536 position format, with every fourth colony position left empty, on a solid nutrient medium with a stressor embedded. Colonies were evolved as ALE lines over 19 growth cycles of 72h. To measure the change in cell doubling time, ALE lineages from each growth cycle were first transferred to preculture plates, with stressors embedded, and non-evolving wildtype colonies were introduced into the empty 4:th positions to serve as spatial controls for environmental variation between colony positions and plates. After a 72 h pre-cultivation the 1152 ALE lines and the 384 spatial controls on each preculture plates were transferred to experimental plates with stressors embedded and cultivated in bench top scanners in thermostatic cabinets, and automated measurements of the light transmitted through each colony were taken at 20 min intervals and converted into estimates of colony population size. Cell doubling times were estimated from the period of maximum growth rate, generations passed were estimated as the number of population doublings and a LOESS fit was made to each resulting adaptation trajectory. The cell doubling time adaptation achieved at 25, 50 and 75 generations was then extracted from the LOESS fit to each trajectory and used in downstream analysis.

**Figure S2. Pre-adaptation cell doubling times of single gene deletion strains on arsenite.** Density distribution of mean cell doubling times of 4639 single gene deletion strains exposed to 0, 1, 2, 3 and 4 mM of arsenite (mean of *n*=3-6 replicates), before adaptive evolution.

**Figure S3. Comparing the cell doubling time adaptation of gene deletion strains to arsenite after 25, 50 and 75 generations of evolution.** Comparing the cell doubling time reductions achieved by 4639 yeast deletion strains (mean of *n*=3-6) exposed to arsenite (3mM) A) over 75 (*y*-axis) vs. 25 generations (*x*-axis) and B) over 50 (*y*-axis) vs 25 generations (*x*-axis). Linear regressions and the squared coefficient of linear regression (also shown in Fig. 1D) are shown.

**Figure S4. Adaptation of gene deletion strains to arsenite is near perfectly predicted by their fitness.** The cell doubling time reduction in 4639 yeast deletion strains adapting to arsenite (3mM) over A) 25 generations and B) 50 generations, as a function of their pre-adaptation cell doubling time. Mean values (*n*=3-6) are shown, wildtypes are indicated with white squares (*n*=384). Genes traditionally held to influence evolvability (blue), and deletion strains with a delayed *ARR3* expression and therefore a delayed physiological adjustment to arsenite, (orange) are marked. The linear regression and the squared coefficient of linear regressions (also shown in Fig 2A) are shown.

**Figure S5. Adaptation of yeast deletion strains to 3 mM As[III] at high replication is near perfectly predicted by initial fitness.** Cell doubling time reductions of 345 yeast deletion strains after A) 25 and B) 50 generations of adaptation to arsenite (3 mM), as a function of their pre-adaptation cell doubling time. Mean values of *n*=12 independently evolving ALE replicates are shown. White squares indicate the wildtype (mean of *n*=468 replicates). Linear regression lines and squared linear regression coefficients (also shown in Fig 3A) are shown.

**Figure S6. Adaptation of yeast deletion strains to 4 mM As[III] is near perfectly predicted by initial fitness.** Cell doubling time reductions in 330 yeast deletion strains after A) 25 and B) 50 generations of adaptation to arsenite (4 mM), as a function of their pre-adaptation cell doubling time. Mean values of *n*=12 independently evolving ALE replicates are shown. White squares indicate the wildtype (mean of *n*=648). Linear regression lines and squared linear regression coefficients (also shown in Fig 3B) are shown.

**Figure S7. Cell doubling time adaptations of gene deletion strain to different environments are uncorrelated.** Comparing the cell doubling time reductions in 330 yeast deletion strains (mean of *n*=12 independently evolving ALE replicates) over 75 generations of adaptation to arsenite 4 mM, paraquat 400 mg/L, rapamycin, 0.25 mg/L and sodium chloride, 1.25 M. Numbers and colors indicate Pearson’s correlation coefficient, *r*, for each pair of environments.

**Figure S8. Adaptation of gene deletion strains to different stresses occurs at a cost of slower growth in absence of stress.** The percentage increase in cell doubling time in absence of stress (SC background medium) in yeast deletion strains (*n*=330-345) adapting over 75 generations to different stresses. Mean of *n*=12-16 replicates were used. Horizontal lines indicate the median, boxes show the interquartile range and whiskers show 1.5x the interquartile range. Violin outlines show the probability distribution.

**Figure S9. Some gene deletion strains adapting significantly slower than expected are shared across environments.** Cell doubling time reductions in 330 yeast deletion strains after A) 25 and B) 50 generations of adaptation to 4 mM arsenite (left panel) and 1.25 M NaCl (right panel), as a function of their pre-adaptation cell doubling time. Mean values of *n*=12 independently evolving ALE replicates are shown. Linear regression lines and squared linear regression coefficients (also shown in Fig 3B) are shown. 14 gene deletion strains with significantly slower adaptation (Students t-test, FDR, *q=*0.05) to both arsenite sand NaCl are indicated in red and named. Blue names indicate four of these gene deletion strains that also adapt significantly slower to 400 mg/L of paraquat.

**Table S1:** List of selection agents used in the evolution experiments

| **Selection** | **Compound** | **Concentration(s)** | **Type** |
| --- | --- | --- | --- |
| Arsenite (III) stress | Sodium arsenite (NaAsO_2_) | 3 and 4 mM | Toxic metalloid |
| Mitochondrial superoxide stress | Paraquat | 400 mg/L | Mitochondrial superoxide producer |
| Rapamycin stress | Sirolimus | 0.25 mg/L | TOR-inhibitor |
| Osmotic stress | Sodium chloride (NaCl) | 1.25 M | Salt |
